# A Focused Review of Neural Recording and Stimulation Techniques With Immune-Modulatory Targets

**DOI:** 10.3389/fimmu.2021.689344

**Published:** 2021-09-27

**Authors:** Lorenzo Carnevale, Marialuisa Perrotta, Giuseppe Lembo

**Affiliations:** ^1^ Research Unit of Neuro and Cardiovascular Pathophysiology, IRCCS Neuromed, Department of Angiocardioneurology and Translational Medicine, Pozzilli (IS), Italy; ^2^ Department of Molecular Medicine, “Sapienza” University of Rome, Rome, Italy

**Keywords:** immunity, bioelectronic medicine, inflammatory reflex, microneurography, neuro-immune interface

## Abstract

The complex interactions established between the nervous and immune systems have been investigated for a long time. With the advent of small and portable devices to record and stimulate nerve activity, researchers from many fields began to be interested in how nervous activity can elicit immune responses and whether this activity can be manipulated to trigger specific immune responses. Pioneering works demonstrated the existence of a cholinergic inflammatory reflex, capable of controlling the systemic inflammatory response through a vagus nerve-mediated modulation of the spleen. This work inspired many different areas of technological and conceptual advancement, which are here reviewed to provide a concise reference for the main works expanding the knowledge on vagus nerve immune-modulatory capabilities. In these works the enabling technologies of peripheral nervous activity recordings were implemented and embody the current efforts aimed at controlling neural activity with modulating functions in immune response, both in experimental and clinical contexts.

## Introduction

How the central nervous system is able to communicate with all the other organs and systems across the living body has always represented an intriguing issue. On the technical side, the challenge has been similar, considering the complexity of the brain to body interactions under examination. While it was quite easy to measure the global neural activity in terms of brain cortical activity through electroencephalogram, or nervous activity directed to the skeletal muscle to control movement, measuring the nerve activity controlling visceral organs through autonomic nervous system (ANS) innervation was challenging. Hence, researchers often exploited the analysis of surrogate markers of nervous system activity, such as neurotransmitter spillover and tissue concentration, or the modulation of vital parameters which were tightly related to the ANS balance (heart rate, respiration rate, and intestinal motility). On this note, although the complexity and invasiveness of surgical approaches necessary to expose the multitude of peripheral nerves of interest make this approach possible in animal models, the translation to humans remains a challenging issue. The great technological improvements in material science and manufacturing, coupled with miniaturization of electronic circuits and devices, gave researchers new tools to characterize, in a direct way, the activity of the nervous system in modulating the connections with peripheral tissues and organs (1, 2).

The investigation of ANS regulation of several physiological systems, such as the cardiovascular and renal districts, prompted the improvement of procedures and equipment necessary to perform microneurography. In this context, the straightforward accession to nerves projected to the organs of interest, like the renal nerve or the carotid baroceptors and the cervical trunk of the vagus nerve, allowed early breakthroughs, which shed light on the mechanisms underlying the interplay between blood pressure regulation, baroreflexes, and nerve activity (3, 4).

The immune system is one of the main regulators of body homeostasis. The first hints suggesting that the immune system is also tightly regulated by the nervous system go back to the beginning of the past century. The various mechanisms by which the neuro-immune interfaces are established in different physiological and diseases contexts are thoroughly described elsewhere (5–7). Connections and crosstalk between nervous and immune systems are established at every endpoint of the peripheral nervous system (PNS), with both somatosensory and ANS’ afferent and efferent arms. For example, the somatosensory system allows receiving stimuli from the immunoinflammatory milieu and communicate them to the CNS through the nociceptors residing in the dorsal root or trigeminal ganglia. On the other hand, the ANS establishes routes of bidirectional communication between the CNS and peripheral organs mainly through noradrenergic and cholinergic nerves. Also, specific immune cells are able to respond to neurotransmitters and, at the same time, secrete them, acting as neural relays (8).

At the beginning of this century a solid body of works pivoting around the connections established between the nervous and immune systems demonstrated the existence of a direct neural control of immunity and inflammation (9, 10). These breakthrough findings paved the way for a new field of research centered on the existence of the neural control of immunity, which led researchers to investigate the existence of direct and/or indirect circuits elicited by the nervous system and capable of driving specific immune responses with a therapeutical and translational outlook. The scope of this review is to provide a concise overview of works focusing on neuro-immune modulation, by which it is possible to analyze and stimulate neural activity to obtain different effects on immune cells activation and possibly modulate inflammatory responses.

Investigating the role of the innervation reaching the immune system, which comprises primary, secondary, and tertiary lymphoid organs, involved in different stages of immune cells maturation and immune responses, is a complex challenge requiring the refinement of electronics, materials, and genetic tools to directly measure nerve activity during homeostasis and diseases. How these challenges were addressed to unravel the neuroimmune mechanisms underlying physiology and pathophysiology will be discussed in section 2 of the current review.

On the other hand, the demonstration of the existence of neuro-immune circuits opened the possibility to identify new therapeutic targets achievable by modulating these circuits with genetic or electronic tools, to control the immune response in a specific way, without the use of pharmacological system-wide modulators, often carrying several undesired and off-target effects. The branch of research aimed at translating these findings to human pathologies, called bioelectronic medicine, has been only recently implemented at the pre-clinical and clinical levels. As this field is only at the beginning of discovering its therapeutic application potential, several potentially interesting unexplored paths and contexts are emerging. The current technologies and results achieved with bioelectronic medicine and mechanistic findings obtained by stimulating nerves in a tightly controlled manner are presented in section 3 of the current review.

## Analyzing Nervous System Activity to Understand Its Modulatory Action on the Immune System

The first efforts focused toward the direct measurement of the interplay established between neural activity and the immune system can be found in the first years of the nineties, when Nijima et al. demonstrated by direct renal, splenic, and adrenal nerve recording that the intravenous injection of IL-1β upregulated splenic nerve activity and suppressed renal nerve activity, while observing a fall in arterial pressure not affected by baroceptor denervation (11). These findings suggested that IL-1β induced a modulation of the splenic nerve activity, directly mediated by the brain, thus allowing them to propose a role of the central drive in eliciting peripheral immune responses. Subsequent works from the same group demonstrated that if IL-1β was injected into the portal vein, it increased the activity of the afferent branch of the hepatic vagus nerve. Also, an increase of splenic nerve activity mediated by a central reflex was observed and interestingly it was hampered by a resection of the hepatic branch of the vagus nerve (12, 13). These studies were hints to the breakthrough discoveries at the dawn of the new century, when Tracey and coworkers demonstrated the existence of the inflammatory reflex, whereby the nervous system was proved capable of regulating peripheral inflammatory responses through the efferent vagus nerve, in a similar way to the control exerted by the ANS on heart rate, respiration, and other vital functions (10). Their work paved the way for a completely new field of research, which shed light on the mechanisms by which the inflammatory reflex exerts its action, and proposed new concepts underlying the interplay between the two systems, demonstrating the existence of immune cells capable of synthetizing neurotransmitters, after being primed by neural signals (8, 14).

In subsequent years, the field of bioelectronic medicine exhibited a sharp increase in terms of applications and technological improvements. Even after acknowledging the initial challenges posed by the necessity of analyzing signals coming from peripheral nerves with high accuracy and fidelity (15), several solutions have been proposed to optimize surgical procedures and set up, paving the way to the possibility of recording neural activity in a uniform manner (16–19).

This wave of technological evolution let researchers use reliable data to decode the neural circuits involved in immunomodulatory processes. Most of the attention has been focused on analyzing one of the main brain connections established with the rest of the body, the vagus nerve, achieving the goal of identifying a characteristic pattern of firing to discriminate whether the mouse under examination was exposed to IL-1β, TNF-α, or no cytokine (20). The vagus has also been investigated as a vector of neural signaling directed toward the gut, which has been shown as one of the fundamental regulators of systemic inflammatory and immune processes (21, 22).

Shifting the attention to other peripheral nerves, the main focus of investigation in the neuro-immune context has been the splenic nerve, identified as one of the modulators of the systemic inflammatory milieu (23). It has been demonstrated that hyperthermia upregulates inflammatory genes expression, and that this effect is mediated by splenic nerve activity (24). In the last ten years, our group demonstrated the fundamental role that the splenic nerve has in priming the immune response to angiotensin II, a hormone peptide which is capable of elevating blood pressure in experimental animals, priming T cells in the spleen and stimulating their egression toward target organs typically characterizing hypertensive damage, like in the vasculature and kidneys (25). An analogous circuit was identified in response to a different hypertensive stimulus, deoxycorticosterone-acetate salt that typically reproduces salt-sensitive hypertension (26). This response was primed by an elevated neural activity which recruited the splenic noradrenergic pathway (27) and was dependent on a direct interface between the celiac branch of the vagus nerve and the splenic nerve. Further investigation of this neural circuit showed that the splenic nerve activity increase was directly induced by an upregulation of the efferent branch of the celiac vagus nerve (28).

**Figure 1:**
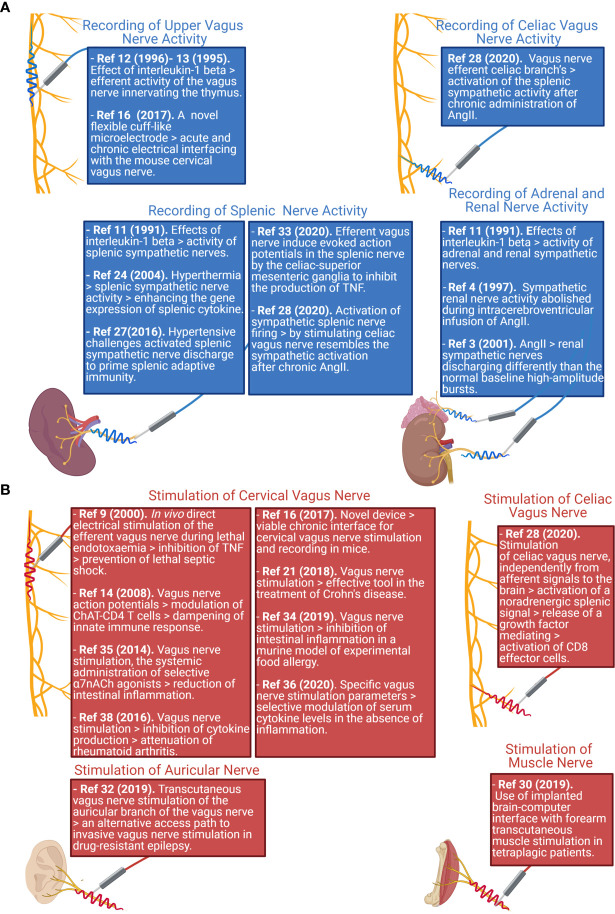
Schematic representation reporting the main nerve recording **(A)** and stimulation **(B)** sites studied in literature, and the associated paper reporting findings on those nerve districts. Created with Biorender.com.

**Figure 2:**
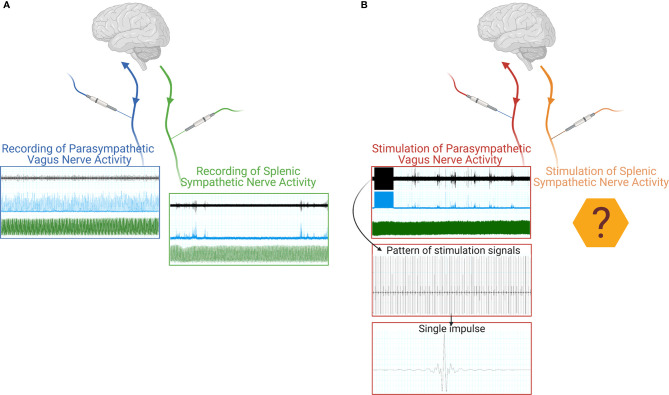
Schematic representation of a neural signal recording **(A)** of both parasympathetic (blue box) and sympathetic nerve activity (green box). The raw neural traces are shown in black, the integrated signals in blue, and the blood pressure recordings in green. Schematic representation of a VNS stimulation **(B)**, the raw neural traces are shown in black, the integrated signals in blue, and the blood pressure recordings in green, with progressive detail on the electrical stimulus train and the single stimulus waveform. Created with Biorender.com.

Since peripheral nerves are heterogeneous in terms of size, surgical approaches, and the types of activity which could be recorded, literature focused on a variety of approaches (**Figure 1**). To provide the reader with an example of the multitude of signals that are transmitted, in **Figure 2A** we show two different nerves and physiological variables recordings: boxed in blue there is an example of a two min recording of the celiac vagus nerve (raw signal in black, integrated signal in blue), characterized by rhythmic neural discharge well coupled with the blood pressure signal (green track). Boxed in green there is an example of a two min splenic nerve recording, characterized by isolated spikes non-synchronous with the blood pressure recordings.

## Eliciting Nervous Activity Through Nerve Stimulation to Alter the Inflammation and Immune Response

In clinical practice, vagus nerve stimulation (VNS) has been proven as an invaluable tool to treat neurological conditions such as epilepsy or psychiatric disorders such as depression (29), whereas peripheral nerve stimulation has been used to directly evoke a muscle contraction in the rehabilitation context (30). These applications have the characteristic of leveraging a closed-loop design, providing immediate stimulation based on a physical readout suggesting the necessity of an action (i.e., a seizure onset in epilepsy or trajectory planning in rehabilitation) (2, 31), Moreover, the recent update in term of stimulatory devices gave clinicians the opportunity to test VNS strategies leveraging cutaneous auricular innervation, through the transcutaneous auricular VNS (tVNS), greatly reducing invasiveness and a series of drawback related to the invasive carotid VNS (32).

The exceptional efforts directed toward vagus nerve recording and signal decoding in animal models has the ultimate aim of delivering a translational approach to the neural control of immunity by means of VNS. In the preclinical research, the technical challenges raised by nerve recording procedures similarly apply to the electrodes designed to stimulate a specific peripheral neural district. A powerful addition to the availability of mouse models is the combination of optogenetic approaches and cre-loxP genetic engineering. In this way it is possible to generate mice in which the optogenetic stimulation selectively recruits specific nerve fibers of choice, making it possible to precisely identify the brain regions from which the neural circuits originated (33).

In animals, the bioelectronic VNS proved effective in downregulating the TNF-α increase observed in response to lipopolysaccharides (LPS) injection (9), providing the first proof of concept of a potential therapeutic strategy for inflammatory systemic diseases. A series of experiments, based on this model, thoroughly explained an immunomodulating mechanism in which LPS stimulated vagus nerve afferent activity, signaling danger to the brain. In the brain, specific areas are activated and recruit downstream nerve activity through the efferent arm of the vagus nerve and transduce this signal to the splenic nerve (33). Here the splenic nerve endings are capable of modulating and regulating lymphocyte functions: in this case they activate a specific T-cell niche which expresses choline acetyltransferase (ChAT). These cells are activated by bioelectronic stimulation of the vagus nerve by noradrenergic signaling and function as a neural relay, starting the biosynthesis of acetylcholine which in turn activates a population of anti-inflammatory macrophages (8).

Leveraging these anti-inflammatory properties, VNS has been tested as a treatment for food allergy (34) and intestinal inflammation (35). However, growing evidence suggests that the vagus nerve may not only be recruited in the context of an anti-inflammatory reflex, but also may be modulated as an inflammatory reflex. In fact, recent works showed that stimulating the celiac branch of the vagus nerve primes a splenic immune response, conveyed by the noradrenergic signaling in the spleen. This signaling recruits an α-adrenergic pathway, which upregulates Placental Growth Factor (PlGF), a key molecular player of the neuro-immune signal transduction (25), previously reported as capable of activating a specific subset of T cells, namely CD8 effector T cells, to promote their egression from the spleen (28). Overall, these data suggest that specific combinations of stimulation current and frequency can alter the cytokine landscape toward pro- or anti-inflammatory responses (36).

The current translation of immune modulation achieved by electronic medicine mainly focused on pathologies in which the recruited effect is anti-inflammatory, like Crohn’s disease, Rheumatoid arthritis, and metabolic syndrome. In these contexts, VNS has been mainly tested in patients refractory to pharmacological therapies, showing promising results with the majority of patients reporting beneficial effects and reduced disease severity (37–39). Most of the clinical studies relying on VNS however face the major limitation of this technique: with the current technology they are not capable of selectively modulating one aspect of immunity and relying on the cervical VNS property of lowering circulating inflammatory cytokines like TNF-α, IL-6, and IL-17 (38), with the main clinical endpoint to lower pathology severity. For a comprehensive review of human studies involving nervous stimulation see (39).

The current body of literature and data available always focuses on the stimulation of the cervical vagus nerve (**Figure 1B**), since it is the easiest to be surgically accessed and is directed to most of the internal organs. As such, the cervical vagus nerve has been identified as an optimal target for bioelectronic stimulation. As an example of this procedure, **Figure 2B** shows, in the red box, a track recording of a stimulation procedure carried out on the celiac branch of the vagus nerve. The signal elicited on the splenic nerve (raw signal in black, integrated signal in blue, blood pressure in green) was concomitantly recorded, highlighting the impulse train and the single impulse provided to the nerve.

## Discussion

The current landscape of the research efforts directed toward the recording and interpretation of neural signals in the context of immune modulation is flourishing with net context and pathologies under scrutiny. Abundant and reliable evidence of the reflex-like regulation of inflammation has subverted the classical duality between sympathetic and parasympathetic autonomic regulation, pushing researchers to investigate the specific regulatory neural circuits driving the immunity in specific contexts. The heterogeneous cultural background of different specialties approaching nerve recording and signal interpretation results in a heterogeneity of procedures and lack of a standard approach. To hamper this problem, research groups, which discovered the inflammatory reflex, proposed methodology and procedures that could help in standardizing the recordings and analysis of the vagus nerve (40).

While preclinical bioelectronic medicine is in optimal shape and a fast-growing field, the translation of the findings to the clinical setting is slow. In fact, if VNS has been shown to be effective for autoimmune diseases such as rheumatoid arthritis and inflammatory diseases like Crohn’s disease, it is not yet under consideration for pathologies where a fine modulation of immune responses could be necessary.

The current technologies let us record nervous activity on several nervous districts, such as the vagus or splenic nerve (**Figure 2A**), and also procedures stimulating the vagus nerve are consolidated. On the other hand, no stimulation has been tested on peripheral sympathetic nerves, except for the musculoskeletal nerves exploited to trigger muscle contraction and thus movement. In principle, it could be feasible to apply a similar concept to peripheral sympathetic nerves to obtain a targeted stimulation to internal organs without soliciting off-target effects due to a nonspecific nerve stimulation (like the one obtained on the cervical vagus nerve) (**Figure 2B**).

In the foreseeable future, the enabling technologies of nerve recording and stimulation, miniaturization of devices, and improved analytical capabilities, such as artificial intelligence, will allow researchers to overcome current limitations in this field and recommend it as a reliable and pervasive alternative to pharmacological treatments. On the clinical side, the main issue is the lack of fast and precise readouts for the design of closed-loop immune-inflammatory control systems. If researchers will be able to provide such readouts, like seizure-onset in the context of epilepsy, it will be possible to fine tune the stimulation pattern and elicit the desired immuno-modulation (36), and a fundamental issue to be resolved in order to precisely target immune mechanisms in human pathologies. In addition, further studies will be needed to identify similar possibilities of modulation for the activation of specific immune cell subsets. In the experimental setting, nerve decoding and stimulation will become more and more prevalent not only as a valid therapeutic proof of concept, but also as an enabling technology to model specific patterns of immune cell activation (28) or inflammatory modulation (36).

Overall, bioelectronic medicine approaches have shown the potential to deeply impact the immune system and modulate it, while a growing number of researchers, both in the field of technology and life sciences, are contributing to expand the possibilities of investigation and combine knowledge in terms of how the nervous system can influence and modulate the immune system. If the different operators involved in this branch of research will be able to cooperate and collaborate to synthesize their findings, their final aim and product will be an atlas of the different nervous activation patterns corresponding to the desired immune system modulation and a series of stimulating devices and tools to directly evoke and elicit them.

## Author Contributions

LC wrote the manuscript. MP drafted figures and schematics. GL provided funding and supervised the work. All authors contributed to the article and approved the submitted version.

## Funding

Italian Ministry of Health “Ricerca Corrente” and ERA-CVD (PLAQUEFIGHT) 01KL1808 to GL.

## Conflict of Interest

The authors declare that the research was conducted in the absence of any commercial or financial relationships that could be construed as a potential conflict of interest.

## Publisher’s Note

All claims expressed in this article are solely those of the authors and do not necessarily represent those of their affiliated organizations, or those of the publisher, the editors and the reviewers. Any product that may be evaluated in this article, or claim that may be made by its manufacturer, is not guaranteed or endorsed by the publisher.
